# End of the Volume

**Published:** 1886-12

**Authors:** 


### END OF THE VOLUME.

With this number Vol. VII is brought to a close. The index for the year is sent out with it, and we commend that to the examination of our readers. The list of original contributors comprises some of the best known names in dentistry, and a study of the titles of the original papers published during the year will show that there are many articles which will take their place in the permanent literature of the profession. The volume is larger than any of its predecessors, and comprises more than 700 pages of reading matter, every line of which was carefully prepared expressly for this journal. Think how much of labor the preparation of such a volume implies, for there has been no reaping of that which another has sown, no reprinting of articles from other sources.

Has not the journal well earned its subscription price? Not one number during the year has been a day behind in its issue. It has been prompt in its appearance, and its subscribers know upon what precise day and by what particular post it may be expected to arrive. Ought it not, therefore, to anticipate equal promptness from those whom it visits? Yet there are a number who are indebted to it, although they must be aware that their subscription money is the only thing that will enable it to continue its appearance. They know that its publishers have no selfish ends to compass, but are honestly and conscientiously trying to serve a profession which they love. They have a right, then, to expect some appreciation of their efforts.

With this number will be sent out bills to those who are indebted for subscriptions, and we ask YOU, reader, if such an one be enclosed in your copy, to remember that we are waiting for that which is justly our own. Send to the Editor at Buffalo, in postal notes or orders, or drafts on New York, what you owe.

And now, what shall we say for the coming year? If the accomplishments of the past will not speak for the journal, it is folly to make promises for the future. We can only say that we honestly believe that Volume VIII will be better than Volume VII. Send your subscription for the new volume and see if it is not.

It is a matter of pride to each one of the publishers that very seldom does any one write asking that his journal be stopped. There is, at least upon our part, a lively affection for every reader, and we earnestly hope that the feeling is mutual. When for some good cause it becomes necessary to drop a name, it seems like parting with a dear friend. May these partings grow continually less.

There is one thing that the Editor desires to impress upon the mind of every reader, and that is the fact that, although his is the name that is pushed to the front by his associates, his is not the credit for all the good work which the journal does. If the profession could know of the unselfish and exhaustive labors that are performed by others of the publishers, of the liberality with which they at times put their hands in their pockets and bring forth for the good of their profession and the journal which they love, but through which they expect to compass no selfish ends, it would convince every dentist that there is good in the world of which he, perhaps, had no knowledge. The Editor of this journal is cognizant of many such acts that could be prompted by none but the noblest sentiments, and he alone knows how much of true, generous, professional feeling has often existed in the turmoil of daily professional life.

Do you think, dear reader, that we are indulging in unwarranted sentiment and basely romancing for an ulterior purpose? You never were more mistaken, if you do. There is a great deal of good in this world which men will not see, and more which is never brought to the light of day. The editor has but been taking into his confidence a class of men who seem very near to him—the subscribers to THE INDEPENDENT PRACTITIONER.

